# Reducing Unplanned Extubations in a Pediatric Intensive Care Unit: A Systematic Approach

**DOI:** 10.1155/2009/820495

**Published:** 2009-12-30

**Authors:** Bonnie R. Rachman, Robin Watson, Norline Woods, Richard B. Mink

**Affiliations:** ^1^Division of Critical Care Medicine, Department of Pediatrics, Harbor-UCLA Medical Center, David Geffen School of Medicine at UCLA, Torrance, CA 90509, USA; ^2^Department of Nursing, Harbor-UCLA Medical Center, Torrance, CA 90509, USA

## Abstract

*Objective*. To prospectively determine the rate of unplanned extubations and contributing factors and determine whether a targeted intervention program would be successful in decreasing the rate of unplanned extubations. *Design*. Prospective, observational study. *Setting*. A 10-bed Pediatric Intensive Care Unit (PICU). *Patients*. All intubated pediatric patients during two time periods: September 1, 2000–March 31, 2001 and November 1, 2001–April 30, 2002. *Interventions*. After determining the rate and causes of unplanned extubation, a program was developed consisting of education and a formalized endotracheal tube taping policy. Data were then collected after implementation of the program. *Measurements and Main Results*. Prior to the implementation of the program, there were 10 (14.7%) unplanned extubations for a rate of 6.4 unplanned extubations per 100 ventilated days. Of the ten unplanned extubations, reintubation was required in 2 (20%). Inadequate sedation, poor taping, and improper position of the endotracheal tube were the items most frequently cited as causing an unplanned extubation. Following the program, there were two (3.4%) unplanned extubations for 1.0 unplanned extubations per 100 ventilated days. Neither patient required reintubation. There were no significant differences (*P* > .05) in age, weight, endotracheal tube size, or duration of intubation in the two time periods. However, there was a significant decrease in both the number (*P* = .03) and the rate (*P* = .04) of unplanned extubations after the implementation of the quality improvement program. *Conclusions*. The rate of unplanned extubation in a PICU can be decreased with a quality improvement program that targets the institution's specific needs.

## 1. Introduction

Improving quality of care in the pediatric intensive care unit (PICU) is an imperative in healthcare. Quality improvement efforts are focused on care processes with the goal of eliminating errors and adverse events. This process begins with the identification of a problem and its causative factors. Then, a plan is implemented to eliminate these factors. The results are analyzed to ascertain whether the plan has decreased the identified problem.

The use of endotracheal intubation is routine in the care of critically ill children [1]. Extubation is performed when the need for mechanical ventilation has resolved. Unplanned extubation is the displacement or removal of the endotracheal tube at a time other than that specifically chosen for a planned extubation and is a serious adverse event [2–4]. Previous investigations have shown that the rate of unplanned extubations in infants and children in the PICU ranges from 0.114 to 4.36 per 100 ventilated days [5, 6]. Generally, 1.0 unplanned extubations per 100 ventilated days are considered within national standards acknowledging that all unplanned extubations are unacceptable [2, 5].

Unplanned extubation exposes the patient to morbidity and mortality over and above those associated with the patient's underlying disease [6, 7]. Kurachek et al. showed that an unplanned extubation prolongs time of intubation thereby increasing the patient's exposure to hazards of airway intervention and mechanical ventilation [2]. In their investigation, PICU length of stay more than doubled after an unplanned extubation. 

It is more common to require reintubation after an unplanned extubation than after a planned extubation [8]. In addition, emergent reintubation may be needed at a time when the patient has not been fasting, posing a risk of aspiration [9]. Moreover, reintubation may be needed when personnel available for the procedure have less experience and skill with emergency airway management in contrast to a reintubation that takes place after a planned extubation where appropriate staff is readily available [2, 10]. In a multicenter study of risk factors and outcomes of extubation failures in the PICU the failure rate after unplanned extubation was 37.5% but only 6.2% after a planned extubation [2]. All unplanned extubations are unacceptable due to their potential for causing unnecessary harm to the patient.

Our impression was that there was a high rate of unplanned extubations in our PICU. As a quality improvement effort, we prospectively determined the unplanned extubation rate in the PICU as well as the contributing factors. Based on these data, we developed a targeted intervention program hypothesizing that it would be able to decrease unplanned extubations.

## 2. Methods

The Institutional Review Board waived the need for informed consent. The study included all intubated patients in a 10-bed PICU located in a general county teaching hospital. The PICU is staffed by board certified pediatric critical care attendants, pediatric critical care fellows as well as pediatric and emergency department residents and interns. At night, care is provided by a senior pediatric resident (PGY 2 or 3) and an intern. The fellow and attendant are available by phone and return to the hospital if needed. Nurse-to-patient ratios are 1 : 1 or 1 : 2 depending on acuity. Nurses administer sedatives and neuromuscular blocking agents as ordered by the physicians. The choice of the particular agent and the dose is based upon the patient's clinical requirements. Sedation protocols are not utilized in the PICU. Physical restraints may be used with a physician order. 

 After intubation, the endotracheal tube is secured with tape, a chest radiograph is performed, and the position of the endotracheal tube is adjusted, if indicated. For patients who are intubated before admission to the PICU, a chest X-ray is obtained as soon as possible after arrival and the tube is adjusted if needed. Although there is no standardized protocol for obtaining radiographs on intubated patients, they are often done on a daily basis. At the time of initiation of the project, there was no standardized policy for taping of the endotracheal tube. 

For the purpose of this study, an extubation was considered to be unplanned when the displacement or removal of the endotracheal tube occurred at a time other than that chosen for a planned extubation. Reintubation was defined as the replacement of the endotracheal tube within 24 hours, regardless of whether the extubation was planned or unplanned. Since both 8- and 12-hour shifts are utilized in the PICU, time periods were arbitrarily defined as 0600–1200, 1201–1800, 1801–0000, and 0001–0559.

Data collected included patient's age, weight, diagnosis, indication for intubation, size of endotracheal tube, and date and time of intubation and extubation. The data were collected by the physician responsible for the patient's care while intubated. For patients who were intubated prior to arrival to the PICU, the time of admission to the PICU was documented as the time of intubation. If a patient was transferred to an outside institution or expired, the time of transfer or death was documented as the time of extubation. These were considered planned extubations. If the extubation was unplanned, the presumed cause was documented by the data collector. Any questions about the cause of the unplanned extubation were discussed with the study investigator who made the final determination. If the patient required reintubation, a new data sheet was started. Each intubation was considered a separate event. 

Data were collected during two time periods. Data gathered during the first time period, September 1, 2000 through March 31, 2001, were analyzed, and the rate and causes of unplanned extubation were determined. A small task force comprised of physician, nursing staff, and respiratory therapy staff was formed to identify specific areas for intervention. An intervention program was developed and subsequently implemented. 

The program was comprised of an education component including a didactic session to improve knowledge about sedation for the intubated patient and the complications of an unplanned extubation. In addition, an endotracheal tube taping policy was developed. This mandated that the endotracheal tube was to be secured by painting the endotracheal tube, upper lip, and cheek with skin adhesive. One piece of pink tape was used to secure the endotracheal tube by placing one end on the right cheek and drawing it across the top lip, pressing firmly for good adhesion. The tape was then wrapped around the tube for a minimum of two revolutions in a clockwise direction. Excess tape was secured to the right cheek. Using a second piece of tape on the left cheek, the procedure was repeated wrapping the tape in a spiral fashion up the tube and back down again. Excess tape was secured to the left cheek. The security of application was tested by gently pulling the endotracheal tube up and away from the patient's face. The tape on the endotracheal tube was required to be completely changed at least every 48 hours or when loose, grossly contaminated, or needed to be repositioned. A detailed procedure was written and a brief computer video was made to illustrate the proper procedure. All nurses and respiratory care practitioners were required to view the video. Competency was demonstrated by correctly performing the proper taping of the endotracheal tube and stating the policy requirements. Prior to this program, unplanned extubations were viewed as a routine part of PICU care. After the implementation of this program, a zero tolerance attitude towards unplanned extubations was adopted.

The effects were evaluated in a second data collection period, November 1, 2001 through April 30, 2002. There were no changes in the use of noninvasive ventilation modalities during the two time periods. Although extensive education about sedation of the intubated patient took place, no sedation protocols were instituted, and the medications continued to be prescribed by physicians. Nursing staff could administer sedative drugs only with a physician order.

In order to standardize the number of intubated days, the unplanned extubation rate per 100 ventilated days was calculated. Ventilator days were calculated using the difference between the times of intubation and extubation in hours and minutes. Ventilated days were only counted for those patients with an endotracheal tube; ventilator days for patients with a tracheostomy were not collected or used in the calculations. Data from the first and second periods were analyzed using Mann-Whitney Utest, Chi-squaretest, or Fisher's exact test, as appropriate. Rates of unplanned extubation per 100 ventilated days for each month of the study were also determined. Data are presented as medians (25th ,75th percentiles), except as noted. Statistical significance was defined at *P* < .05.

## 3. Results

During the initial period, there were 68 intubations in 62 patients (Table 1). Patients were intubated for airway protection (50%), apnea (24%), and respiratory failure (19%). Those patients intubated for airway protection included surgical patients but these data were not specifically gathered. There were 10 (14.7%) unplanned extubations for a rate of 6.4 unplanned extubations per 100 ventilated days. Of the ten unplanned extubations, reintubation was required in 2 (20%). One patient had two unplanned extubations. 

Of the 10 unplanned extubations in the initial part of the study, five happened between 0600–1200, two between 1201–1800, two between 1801–0000, and one between 0001–0559. In the second time interval, one occurred in the 1801–0000 time period and the other occured between 0001–0559.

Inadequate patient sedation, poor taping where the endotracheal tube is not properly secured to the face or “slips” through the tape, improper position of the endotracheal tube either above the clavicles or at or below the carina, and unknown were the items most frequently cited as leading to an unplanned extubation (Table 2). Based on these findings, a targeted intervention program was developed to address these specific issues. 

The program was instituted in September 2001 and training was completed in October 2001. Following the intervention program, there were 59 intubations in 59 patients (Table 1). The patients were intubated for respiratory failure (49%), airway protection (36%), and apnea (8%). In the second period, there were two (3.4%) unplanned extubations for 1.0 unplanned extubations per 100 ventilated days. Neither patient required reintubation. 

When comparing the two time periods, age, weight, endotracheal tube size, and duration of intubation were similar (*P* > .05). There was no difference (*P* > .05) in the use of cuffed endotracheal tubes in the first time period (32% of patients) compared with that in the second period (42%). In addition, there were no changes in personnel or assignments in the two periods. However, there was a difference in the reasons for intubation between the two groups for respiratory failure and apnea. 

There was no apparent increase or decrease in the monthly rate of unplanned extubations prior to the institution of the intervention program (Table 3). Due to the low number of unplanned extubations (*n* = 2), there were insufficient data to perform process control [11]. There was a significant decrease in both the number (*P* = .03) and the rate (*P* = .04) of unplanned extubations after the implementation of the quality improvement program. The ratio of the incidence rate of unplanned extubations before and after the intervention program was 0.15 with a 95% confidence interval of 0.04–0.59. This indicates that the postintervention rate is not greater than 59% and not less than 4% of what it was in the preintervention period.

## 4. Discussion

The ultimate goal of every intervention is to improve the health and quality of life in all patients. The objective of this study was to improve the quality of care in our PICU by reducing unplanned extubations. In order to accomplish this, we used the plan (P) do (D) study (S) act (A) model [12]. PDSA is a dynamic, continuous quality improvement plan. In this process, effective interventions should be aimed at specific features of a target group, and the healthcare problem must be quantifiable. In our case, the target group included all those responsible for the care of the intubated patient in the PICU. The objective was to reduce the rate of unplanned extubations to a level within national benchmark standards. By using this approach, we were able to significantly reduce the unplanned extubation rate in our PICU.

The first stage of PDSA is the planning (P) stage in which there is analysis of the intended area of improvement. In this case, it was determined that the rate of unplanned extubations in the PICU was well above national benchmark standards.

At the same time that we determined the rate of unplanned extubations in, we also examined possible causes. Several factors that contribute to an unplanned extubation have been previously identified [1, 10, 13]. These include inadequate sedation, the use of neuromuscular blockade, improper use of restraints, improper tube position, inattentive support staff who dislodges the tube during routine care (e.g., obtaining a radiograph), inadequate taping of the endotracheal tube, patient-to-nurse ratios of greater than 1 : 1, occurrence of a procedure or transport at the time of the unplanned extubation, and a lax attitude towards an unplanned extubation [1, 10, 13]. As we investigated the causes of unplanned extubation in the PICU during the planning (P) stage, we found that three of the aforementioned factors contributed significantly to the high rate of unplanned extubations. Therefore, the intervention program used in the do (D) phase focused on addressing these issues, and time and effort were not wasted on “correcting” factors that were not contributing to the problem in our unit. In other institutions, different factors may be operative and would need to be addressed in a program specific to that setting.

In the study (S) phase, we recollected data to determine whether the changes achieved the desired results. Using the targeted intervention program, we were able to reduce the unplanned extubation rate from 6.4 to 1.0 unplanned extubations, per 100 ventilated days. When examining the time of day in which the unplanned extubations occurred, the intervention reduced unplanned extubations in all time periods. Nonetheless, even after education about sedation of the intubated pediatric patient, inadequate sedation continued to be a contributing factor in unplanned extubations. Clearly, improved sedation contributed to the decrease in unplanned extubations but since both unplanned extubations in the second time period were attributed to inadequate sedation, the education program was not completely effective. 

 Assessing the level of sedation in the intubated pediatric patient is difficult. Sedation assessment scales such as the Ramsay scale, modified Ramsay sedation protocol, and the COMFORT scale have been used in the assessment of sedation in intubated children as well as for guiding medication administration [10, 14–17]. Only the COMFORT scale has been validated in children [15]. Given that inadequate sedation continued to be a factor, further examination and adoption of a sedation protocol may be helpful. Since care in our PICU does not include the routine use of physical restraints, these data were not examined.

Because our interventions were successful, we acted (A) on them by adopting them on a permanent basis. Nonetheless, we must be careful in attributing our success to our interventions. Some would argue that with the small sample size, the improvement in rate of unplanned extubation was due to the Hawthorne effect [18] where performance improvement is attributed to the fact that performance is being studied and not actual quality improvement. Because PDSA is a dynamic process, the rate of unplanned extubation will be reexamined at a later date to determine whether the level of improvement has been maintained.

Ideally, the statistical process control method would have been used to investigate trends in the rate of unplanned extubation prior to the implementation of the program. However, since there were only ten unplanned extubations in the first time period and two in the second period, this method could not be utilized. Nonetheless, there was no indication that the rate of unplanned extubations had begun to decrease prior to the implementation of the program (Table 3).

The time periods chosen for the study were similar in both groups. They were carefully selected due to the seasonality of pediatric diseases such as respiratory syncytial virus. The six-month period when there were no data collection was to allow for this seasonality. The age, weight, size of endotracheal tube, and duration of intubation were not different in the groups. Although there were differences in the reasons for intubation in the two groups, the differences likely would have biased the results towards a higher rate of unplanned extubation in the postintervention group since the patients intubated for respiratory failure would likely have more secretions and be more ill than those intubated for apnea. The similarity in the two groups leads us to believe that the decrease in the rate of unplanned extubation was due to our interventions and not due to differences in patient groups.

In conclusion, we demonstrated that the rate of unplanned extubation in a PICU can be decreased with a targeted intervention program tailored for the specific problems. This illustrates that efforts directed at improving quality of care should be based on the issues operative at that institution. By doing so, providers will be able to decrease the rate of unplanned extubations in their PICU.

## Figures and Tables

**Table 1:** Clinical features of intubated children before and after the intervention program.

	Before intervention program	After intervention program	*P* value
*N*	68	59	
*Age (months)*	33 (4, 67)	28 (6, 81)	.55
*Weight (kilograms)*	11 (6, 23)	15 (7, 31)	.28
*Endotracheal tube size (mm)*	4.5 (4.0, 5.5)	4.5 (4.0, 5.5)	.54

*Reason for intubation*			
Airway protection	34	21	.07
Respiratory failure	13	29	.003
Apnea	16	5	.02
Upper airway obstruction	5	1	.14
Cardiopulmonary resuscitation	0	1	.46
Hyperventilation	0	1	.46
Other	0	1	.46

*Duration of intubation (hours)*	36 (8,78)	52 (19,141)	.08
*Number of unplanned extubations*	10	2	.03
*Rate of unplanned extubations/100 ventilated days*	6.4	1.0	.04

**Table 2:** Reasons for the unplanned extubation.

	Before intervention program	After intervention program
Inadequate patient sedation	6	2
Inadequate taping of endotracheal tube	2	0
Improper position of endotracheal tube	1	0
Unknown	1	0

**Table 3:** Rate of unplanned extubations/100 ventilated days by study month.

Month	Rate* of unplanned extubations before intervention program	Rate* of unplanned extubations after intervention program
1	0	0
2	2.39	0
3	0.54	0.92
4	0	0.15
5	0.36	0
6	1.21	0
7	1.06	—

*per 100 ventilated days.
